# Analysis of non-small cell lung cancer with miliary lung metastasis in patients harboring epidermal growth factor receptor mutations

**DOI:** 10.1038/s41598-022-23195-9

**Published:** 2022-10-28

**Authors:** Ming-Hung Chang, Kuo-Hwa Chiang, Jiunn-Min Shieh, Kuo-Chen Cheng, Chung-Han Ho

**Affiliations:** 1 Division of Chest Medicine, Department of Internal Medicine, Chi Mei Medical Center, Chiali, Tainan, Taiwan, ROC; 2 Division of Chest Medicine, Department of Internal Medicine, Chi Mei Medical Center, No.901, Zhonghua Rd., Yongkang Dist., Tainan City, 71004 Taiwan, ROC; 3 Department of Medical Research, Chi-Mei Medical Center, Tainan, Taiwan, ROC

**Keywords:** Cancer, Oncology

## Abstract

Miliary lung metastasis is a unique feature of lung metastasis in non-small cell lung cancer (NSCLC), indicating hematogenous dissemination. Some studies reported more frequency of epidermal growth factor receptor (EGFR) mutation and worse prognosis in these patients. Cases were identified from Chi-Mei medical center cancer registry for the period 2015–2019. Inclusion criteria were NSCLC with contra-lateral lung metastasis harboring EGFR mutation, under tyrosine kinase inhibitor (TKI) prescription. Patients with miliary or non-miliary lung metastasis were enrolled for survival analysis. 182 NSCLC patients were enrolled for assessing time to discontinuation of TKI (TD-TKI), progression-free survival (PFS) and overall survival (OS). 54 patients with miliary lung metastasis had average 13.2 months [95% confidence interval (CI) 10.7–15.6] of TD-TKI, 11.4 months (95% CI 9.3–13.6) of PFS, and 21.3 months (95% CI 16.8–25.8) of OS, which were shorter than non-miliary group with marginally statistical significance. In multivariate analysis, miliary lung metastasis had no statistical significance, and other strong prognostic indicators were found including performance status, liver metastasis, EGFR type, and generation of TKI. In NSCLC patients harboring EGRF mutation under TKI prescription, miliary lung metastasis was not a dominant indicator for outcomes evaluation.

## Introduction

Lung cancer is the leading cause of cancer-related deaths, accounting for 18% of all cancer deaths in the world^1^. However, the prognosis of advanced non-small cell lung cancer (NSCLC) has improved because of recent development of different targeted therapies to epidermal growth factor receptor (EGFR) mutation, anaplastic lymphoma kinase (ALK) fusion etc. Nowadays, tyrosine kinase inhibitor (TKI) has been standard of care for initial management of EGFR mutation.

In advanced stage of lung cancer, the lung is frequently a metastatic organ and chest radiography present several different patterns, including multiple pulmonary nodules, lymphangitis carcinomatosis, malignant pleural effusions and enlarged lymph nodes^2,3^. In some cases, numerous diffuse-distributed micro-nodules throughout bilateral lungs, which some investigators have termed miliary intrapulmonary carcinomatosis (MIPC)^4^, miliary lung metastasis or snowstorm sign, indicate hematogenous dissemination of malignancy and differ from cannonball metastases^5^. The most common lung cancer presenting this unique radiologic pattern was adenocarcinoma, and it was highly associated with EGFR mutation^4,6^. In most previous studies, miliary lung metastasis in NSCLC patients harboring EGRF mutations revealed poorer prognosis in most previous studies^4,7–9^. In this research, we investigated the clinical characteristics and prognosis for NSCLC patients with miliary lung metastasis at initial diagnosis.

## Materials and methods

### Study design and patients

This study was a retrospective, single-center, observational study performed at Chi Mei Medical Center in Taiwan. The study was conducted ethically in accordance with the World Medical Association Declaration of Helsinki and was approved by the Institutional Review Board (IRB) of Chi Mei Medical Center, Taiwan (IRB No. 11011-007) and was exempted from informed consent requirements owing to its retrospective design. This study was conducted in patients who were diagnosed with NSCLC from January 2015 to December 2019 through the Cancer Registry at the Chi-Mei Medical Center in Tainan, Taiwan. Patients with clinical distant metastasis (cM1a and above) were enrolled that was classified by eighth edition of the American Joint Commission on Cancer (AJCC) TNM staging system for NSCLC. The definition of elders was the patients aged 65 years or older.

### Imaging

**Figure 1:**
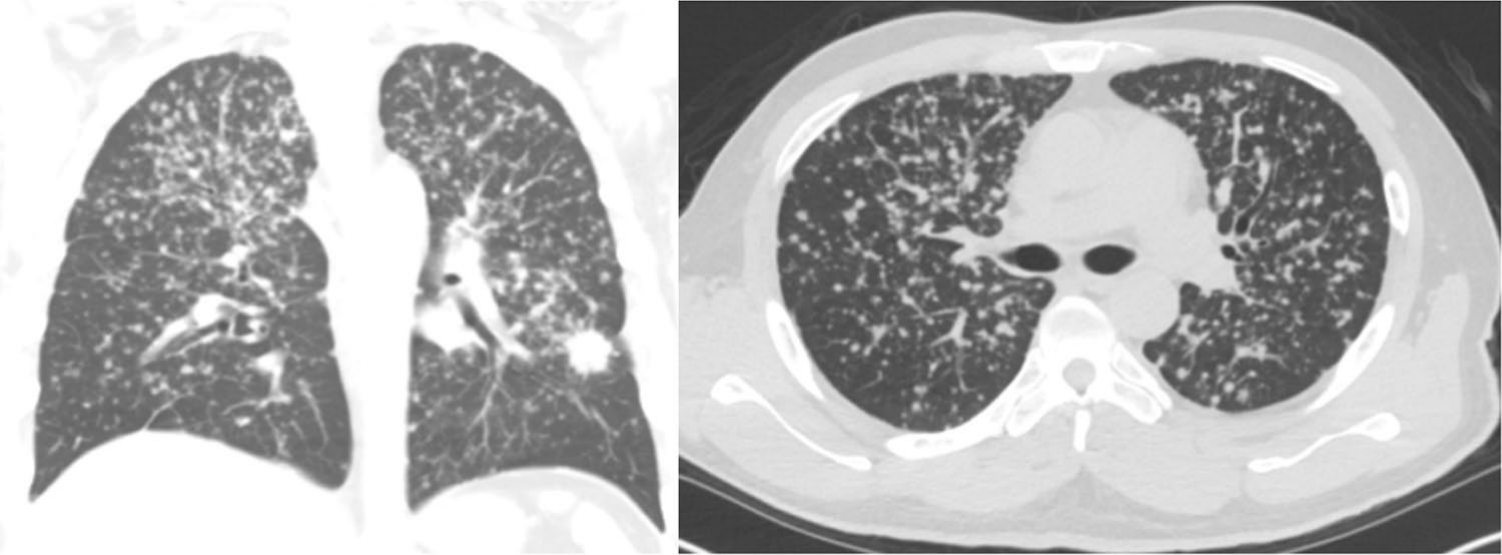
Examples of NSCLC patient with miliary lung metastasis.

The whole-chest computed tomography (CT) was ranged from the level of the superior aperture of the thorax to the upper abdomen containing integral liver. Patients with contra-lateral lung metastasis were confirmed by the report of a radiologist according to typical characteristic, reasonable change with target lesions after therapy by following CT image or histologic tissue proof by further biopsy. Satellite nodules and ipsilateral lung metastasis were excluded. The definition of miliary lung metastasis were: (1) tiny and round pulmonary nodules and most of the nodules < 5 mm in diameter; (2) number of nodules not easily counted and at least over 20; (3) bilateral-lung, discrete, diffuse, random distribution^4^. The examples of miliary lung metastasis are shown in Fig. 1. For comparison, non-miliary lung metastasis indicated single or few solid nodules, multiple uniform nodules with larger size, semisolid nodules, ground-glass opacities or lymphangitic carcinomatosis. In addition, the follow-up intervals of chest CT for treatment response evaluation varied from 2 to 12 months.

### EGFR examination

Tumor specimens from primary lung tumors, metastatic sites or malignant effusion cell blocks were obtained for polymerase chain reaction (PCR) assay and EGFR mutation analysis. EGFR RGQ PCR kit V2 utilized two technologies, ARMS and Scorpions, for detection of mutations in real-time PCR. Specimens were screened for exon 19 deletion mutation, exon 21 L858R point mutation and other uncommon mutations such as exon 20 insertion mutation, G719X, T790M, S768I, L861Q point mutation and complex heterozygous mutation. Uncommon mutations were excluded from outcomes statistical analysis.

### Treatment protocol

First-generation EGFR-TKI, Gefitinib (250 mg) and Erlotinib (100 and 150 mg) and second-generation TKI, Afatinib (30 and 40 mg) were prescribed orally once per day. Some cases received previous chemotherapy with incomplete course for pending EGFR report and some cases adjusted the frequency of TKI to once per 2 to 3 days or 5 times per week by a clinical physician due to side effect. The TKI must be initial first-line systemic therapy. Subsequent use or different TKI switches because of adverse event intolerance or other clinical condition were excluded from outcomes statistical analysis.

### Outcome measures

The endpoints of this study were time to discontinuation of TKI (TD-TKI), progression free survival (PFS) and overall survival (OS). The observation period was 5 years. The TD-TKI was defined as the period from the initiation of the TKI to the date of discontinuation of TKI by any clinical reason such as progression disease (PD), side effect, patient’s decision or hospice intervention. The PFS was defined as the period from the initiation of the TKI to the date of PD or death from any cause. The OS was defined as the period from the initiation of the TKI to the date of death from any cause. The objective response rate was calculated in accordance with RECIST criteria version 1.1 and the PD was defined as an at least 20% increase or an absolute increase of at least 5 mm in the sum of diameters of target lesions, or new metastasis.

### Statistical analysis

All analyses were performed using the statistical software SPSS 24.0 (SPSS Inc., Chicago, IL, USA). All categorical variables were analyzed by the Chi-squared test, except those with an expected frequency of less than five, which were analyzed by Fisher’s exact test. Survival curves were plotted using the Kaplan–Meier method and compared between groups using the log-rank test or generalized Wilcoxon test. Univariate and multivariate analyses calculated crude hazard ratio (cHR) and adjusted hazard ratio (aHR) by Cox proportional hazard model. All *p*-values were based on a two-tailed hypothesis, and statistical significance was assumed if *p* value < 0.05.

### Ethics approval

This study was approved by the Institutional Review Board of the Chi-Mei Medical Center (approval number 11011-007).

## Results

### Basic data

From January 2015 to December 2019, there were 667 NSCLC patients with clinical distant metastasis by 8th AJCC TNM staging system from cM1a to cM1c registered in Cancer Registry. According to chest CT, 401 patients presented with contra-lateral lung metastasis at initial diagnosis. In histological study, the number of miliary lung metastasis was 79 (23.0%) in adenocarcinoma, 7 (16.3%) in squamous cell carcinoma and 1 (7.1%) in NSCLC not otherwise specified. Incidence of miliary lung metastasis had no statistical significance in different histological type of NSCLC (*p* value = 0.278). In extra-pulmonary metastasis survey, the most frequent distant metastasis sites were bone and brain. Higher chance of extra-pulmonary metastasis was found in patients with miliary lung metastasis than in non-miliary group, in percentage of 73.3% versus 44.9% in bone metastasis, *p* value < 0.001; 41.2% versus 26.9% in brain metastasis, *p* value = 0.011; 29.9% versus 16.6% in liver metastasis, *p* value = 0.006, respectively. Among the patients with contra-lateral lung metastasis, EGFR mutation testing was performed for 357 patients with adequate tissue samples and appropriately clinical condition. There were 238 (66.7%) patients were confirmed to harbor EGFR mutations. Patients with miliary lung metastasis had higher rate of EGFR mutation than non-miliary group (78.8% versus 62.9%, *p* value = 0.006).

**Figure 2:**
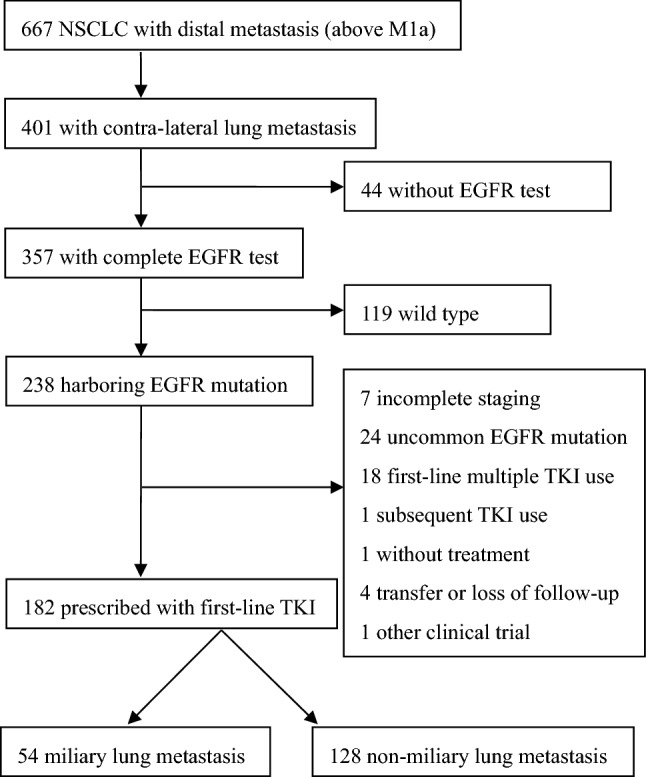
Flow chart of patients screening.

Among the patients with contra-lateral lung metastasis harboring EGFR mutation, there were 182 patients enrolled in the outcome statistical analysis with EGFR-TKI prescription as initial systemic therapy, including 54 patients with miliary lung metastasis and 128 patients with non-miliary lung metastasis. 7 cases with incomplete staging for metastasis evaluation, 24 cases with EGFR uncommon mutations, 18 cases with different TKI switches, one case receiving chemotherapy as initial therapy, one case without treatment, 4 cases with transfer to other hospital or loss of follow-up, and one case with enrollment of other clinical trial, were excluded from the statistical analysis. The flow chart of patients screening is shown in Fig. 2.

In these 182 patients treated with first-line TKI during the study interval, the average/median age was 68.0/68 years, and 116 (63.7%) patients were elders. 62 (34.1%) patients were male. There were 35 (19.2%) smokers. In histological study, most cases were adenocarcinoma, and only one case was adenosquamous carcinoma, one case was NSCLC not otherwise specified. In EGFR mutation analysis, there were 82 cases with exon 19 deletion mutation and 100 cases with L858R point mutation. Gefitinib, erlotinib or afatinib were administered as single TKI therapy in 81 (44.5%), 32 (17.6%) and 69 (37.9%) patients respectively. Overall patient characteristics are shown in Table 1.

**Table 1:** Clinical characteristics of the NSCLC patients with contra-lateral lung metastasis harboring EGFR mutation.

	All patients	Non-miliary	Miliary	*p* value
	n = 182	n = 128	n = 54	
Elder (≥ 65); n (%)	116 (63.7)	87 (68.0)	29 (53.7)	0.068
Male; n (%)	62 (34.1)	42 (32.8)	20 (37.0)	0.583
Smoker; n (%)	35 (19.2)	24 (18.8)	11 (20.4)	0.800
**PS (ECOG); n (%)**				0.032*
0–1	122 (67.0)	92 (71.9)	30 (55.6)	
2–4	60 (33.0)	36 (28.1)	24 (44.4)	
Bone metastasis; n (%)	95 (52.2)	54 (42.2)	41 (75.9)	< 0.001*
Brain metastasis; n (%)	64 (35.2)	39 (30.5)	25 (46.3)	0.041*
Liver metastasis; n (%)	28 (15.4)	16 (12.5)	12 (22.2)	0.097
**EGFR mutation; n (%)**				0.827
L858R	100 (54.9)	71 (55.5)	29 (53.7)	
19 deletion	82 (45.1)	57 (44.5)	25 (46.3)	
**TKI generation; n (%)**				0.398
1st	113 (62.1)	82 (64.1)	31 (57.4)	
Gefitinib	81 (44.5)	55 (43.0)	26 (48.1)	
Erlotinib	32 (17.6)	27 (21.1)	5 (9.3)	
2nd afatinib	69 (37.9)	46 (35.9)	23 (42.6)	
**Response to TKI; n (%)**				
PR	112 (61.5)	81 (63.3)	31 (57.4)	
SD	38 (20.9)	26 (20.3)	12 (22.2)	
PD	21 (11.5)	12 (9.4)	9 (16.7)	
NE	11 (6.0)	9 (7.0)	2 (3.7)	
**Acquired T790M; n (%)**				
Yes	25 (13.7)	17 (13.3)	8 (14.8)	
No	25 (13.7)	18 (14.1)	7 (13.0)	
NE	132 (72.5)	93 (72.7)	39 (72.2)	
TD-TKI; n (%)	162 (89.0)	111 (86.7)	51 (94.4)	0.128
PFS; n (%)	168 (92.3)	116 (90.6)	52 (96.3)	0.190
OS; n (%)	145 (79.7)	98 (76.6)	47 (87.0)	0.109

PS: performance status, ECOG: Eastern Cooperative Oncology Group, PR: partial response, SD: stable disease, PD: progression disease, NE: not evaluable, *p value < 0.05.

### Clinical outcomes between miliary and non-miliary group

**Figure 3:**
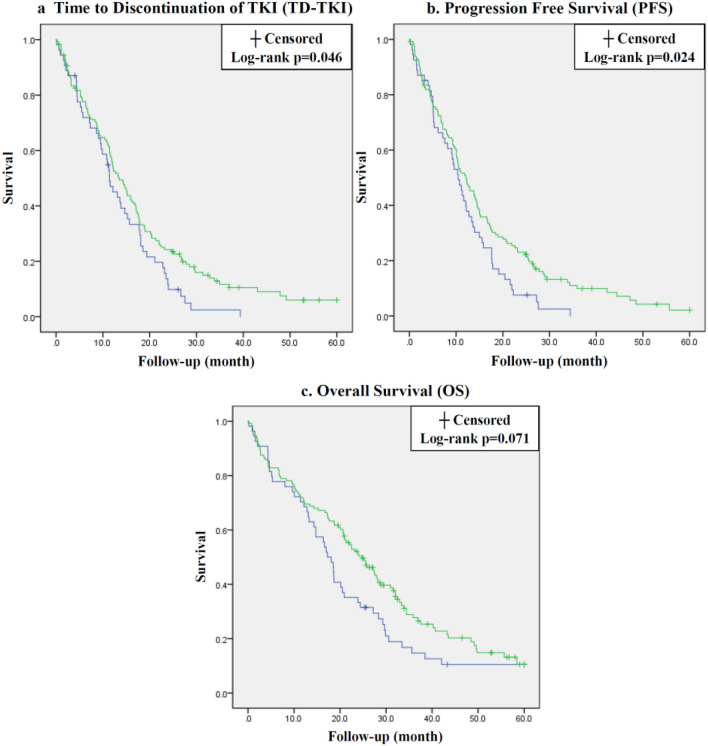
Time to discontinuation of TKI (**a**), progression-free survival (**b**) and overall survival (**c**) curves of patients with miliary (blue line) and non-miliary (green line) lung metastasis.

These 182 patients with contra-lateral lung metastasis harboring EGFR mutation under first-line TKI prescription were admitted for assessing TD-TKI, PFS and OS. All prognosis in the entire patients were 16.4 months [95% confidence interval (CI) 14.3–18.5] in TD-TKI, 14.8 months (95% CI 12.8–16.7) in PFS and 25.1 months (95% CI 22.4–27.9) in OS. Comparison analysis between miliary and non-miliary lung metastasis was done by Kaplan–Meier method shown in Fig. 3. TD-TKI were 13.2 months (95% CI 10.7–15.6) and 17.8 months (95% CI 15.0–20.6) in patients with miliary and non-miliary group (*p* value = 0.046). PFS were 11.4 months (95% CI 9.3–13.6) and 16.2 months (95% CI 13.6–18.8) in miliary and non-miliary group (*p* value = 0.024). OS were 21.3 months (95% CI 16.8–25.8) and 26.8 months (95% CI 23.3–30.2) in miliary and non-miliary group (*p* value = 0.071).

Univariate analysis by Cox proportional hazard model identified miliary lung metastasis (in TD-TKI and PFS), poor performance status (in TD-TKI, PFS and OS), bone metastasis (in OS), brain metastasis (in PFS), liver metastasis (in TD-TKI, PFS and OS) and L858R mutation (in OS) as prognostic indicators for poor outcomes. Multivariate analysis by Cox proportional hazard model identified poor performance status (in TD-TKI, PFS and OS) (Tables 2, 3, 4), liver metastasis (in TD-TKI, PFS and OS) (Tables 2, 3, 4), and L858R mutation (in OS) (Table 4) as independent and strong prognostic indicators for poor survival. Second-generation TKI had significantly better survival than first-generation TKI in all outcomes.

**Table 2:** Associations between miliary and TD-TKI.

	cHR (95% CI)	*p* value	aHR (95% CI)	*p* value
Age (ref = < 65)	1.00 (0.72, 1.37)	0.979	0.92 (0.64, 1.30)	0.629
Gender (ref = female)	0.90 (0.65, 1.25)	0.536	0.78 (0.50, 1.21)	0.264
Smoking (ref = no)	1.16 (0.79, 1.71)	0.454	1.31 (0.78, 2.21)	0.314
PS (ECOG) (ref = 0–1)	2.12 (1.52, 2.95)	< 0.001*	2.06 (1.42, 2.97)	< 0.001*
Bone metastasis (ref = no)	1.33 (0.97, 1.82)	0.073	1.04 (0.73, 1.50)	0.824
Brain metastasis (ref = no)	1.28 (0.93, 1.77)	0.137	1.23 (0.86, 1.75)	0.261
Liver metastasis (ref = no)	2.02 (1.32, 3.11)	0.001*	2.02 (1.26, 3.23)	0.003*
EGFR (ref = 19 deletion)	1.28 (0.94, 1.75)	0.121	1.16 (0.83, 1.63)	0.384
TKI (ref = 1st generation)	0.70 (0.50, 0.96)	0.027*	0.63 (0.44, 0.88)	0.008*
Miliary (ref = no)	1.41 (1.01, 1.97)	0.047*	1.08 (0.73, 1.60)	0.711

**Table 3:** Associations between miliary and PFS.

	cHR (95% CI)	*p* value	aHR (95% CI)	*p* value
Age (ref = < 65)	0.94 (0.68, 1.29)	0.687	0.80 (0.56, 1.14)	0.209
Gender (ref = female)	0.94 (0.68, 1.29)	0.685	0.80 (0.52, 1.22)	0.296
Smoking (ref = no)	1.12 (0.77, 1.64)	0.556	1.18 (0.70, 1.97)	0.535
PS (ECOG) (ref = 0–1)	2.30 (1.66, 3.20)	< 0.001*	2.33 (1.61, 3.37)	< 0.001*
Bone metastasis (ref = no)	1.27 (0.94, 1.72)	0.126	0.97 (0.68, 1.38)	0.869
Brain metastasis (ref = no)	1.47 (1.06, 2.04)	0.020*	1.41 (0.99, 2.02)	0.058
Liver metastasis (ref = no)	1.96 (1.29, 2.99)	0.002*	2.22 (1.40, 3.53)	0.001*
EGFR (ref = 19 deletion)	1.34 (0.98, 1.82)	0.064	1.25 (0.90, 1.75)	0.187
TKI (ref = 1st generation)	0.68 (0.49, 0.93)	0.015*	0.59 (0.42, 0.83)	0.003*
Miliary (ref = no)	1.47 (1.05, 2.05)	0.025*	1.11 (0.75, 1.63)	0.612

**Table 4:** Associations between miliary and OS.

	cHR (95% CI)	*p* value	aHR (95% CI)	*p* value
Age (ref = < 65)	1.48 (1.04, 2.09)	0.028*	1.22 (0.83, 1.80)	0.309
Gender (ref = female)	1.11 (0.79, 1.56)	0.553	1.11 (0.71, 1.75)	0.646
Smoking (ref = no)	1.19 (0.79, 1.79)	0.406	1.15 (0.67, 1.95)	0.620
PS (ECOG) (ref = 0–1)	2.90 (2.05, 4.10)	< 0.001*	2.54 (1.74, 3.71)	< 0.001*
Bone metastasis (ref = no)	1.57 (1.13, 2.18)	0.008*	1.32 (0.91, 1.91)	0.145
Brain metastasis (ref = no)	1.32 (0.94, 1.85)	0.105	1.28 (0.88, 1.87)	0.201
Liver metastasis (ref = no)	2.19 (1.40, 3.44)	0.001*	2.15 (1.33, 3.50)	0.002*
EGFR (ref = 19 deletion)	1.63 (1.16, 2.29)	0.005*	1.57 (1.09, 2.28)	0.017*
TKI (ref = 1st generation)	0.68 (0.48, 0.96)	0.029*	0.62 (0.42, 0.90)	0.011*
Miliary (ref = no)	1.38 (0.97, 1.95)	0.073	1.11 (0.75, 1.65)	0.597

ref: reference, PS: performance status, ECOG: Eastern Cooperative Oncology Group, CI: confidence interval, cHR: crude hazard ratio, aHR: adjusted hazard ratio, *p value < 0.05.

Tables 2, 3, 4 prognostic significance for survival in Cox proportional hazards model of the NSCLC patients with contra-lateral lung metastasis harboring EGFR mutation.

## Discussion

In prevalence, about 13.0% (87/667) of the NSCLC patients with clinical distant metastasis presented with miliary lung metastasis at initial diagnosis in our study. For comparison, about 2.4% (85/3612) of the NSCLC patients from June 2004 to December 2008, presenting with MIPC pattern was reported by Wu^4^. Hematogenous dissemination as miliary lung metastasis also represented higher chance of extra-pulmonary metastasis, in percentage of 73.3% with bone metastasis, 41.2% with brain metastasis and 29.9% with liver metastasis, compared with non-miliary lung metastasis in our study. Wu SG et al. reported that most frequent distant metastasis sites were bone, brain, and liver in NSCLC patients with MIPC pattern, with significantly higher incidence than patients without MIPC. Fu et al. also described that brain and leptomeningeal metastases were more common in diffuse lung metastases group with marginally statistical significance^4,9^. In histological analysis, the most common cell type with miliary lung metastasis was adenocarcinoma (90.8%) in our result, as was like other studies.

Strong association between milary lung metastasis and EGFR mutation was reported by different studies. Togashi Y et al. found that among patients with pulmonary metastases, 11 of 22 patients (50%) harboring EGFR mutations had miliary pulmonary metastasis compared with the presence of miliary pulmonary metastasis in 4 of 33 (12.1%) patients with wild-type EGFR (*p* value = 0.0043), and Wu et al. reported that advanced NSCLC patients with MIPC had a higher EGFR mutation rate than patients without MIPC (70% versus 56%, *p* value = 0.036)^4,6^. In EGFR mutation type analysis, Wu et al. and Kim et al. showed higher rate of exon 19 deletion mutation in miliary lung metastasis^4,7^. However, Okuma et al., Fu et al. and our study all revealed no significant difference of incidence between exon 19 deletion mutation and L858R mutation in different pattern of lung metastasis^8,9^. In our study, the incidence of acquired T790M mutation after PD was analyzed by re-biopsy or plasma screening. Previous 19 deletion mutation had higher rate of acquired T790M mutation than previous L858R mutation (69.6% verse 33.3%, *p* = 0.011). No significant difference in incidence of acquired T790M mutation was found between miliary lung metastasis and non-miliary group (53.3% verse 48.6%, *p* = 0.785).

High rate of EGFR mutation implies that TKI is better choice of treatment than chemotherapy. Common mutation of EGFR such as exon 19 deletion and L858R point mutation and other rare mutation such as exon 19 insertion, point mutation of S768I, L861Q and G719X, are associated with responsiveness to EGFR TKI therapy. Otherwise, exon 20 insertion mutation except for some specific sequence is associated with lack of response to TKI, and T790M point mutation is most observed as a mutation that arises in response to and as resistance mechanism to first- and second-generation of TKI^10^. Response to TKI in mutated EGFR metastatic lung adenocarcinoma was associated with maximum of standardized uptake value (SUVmax) of primary tumor in 18F-fluorodeoxyglucose positron emission tomography (FDG PET), serum CEA level, gender, and smoking history^11^.

Hsu F et al. described that the presence of miliary metastases did not predict for poor overall survival^12^. However, other studies present poorer prognosis in patient with miliary pulmonary dissemination, especially in those harboring EGFR mutations under first-generation TKIs (Gefitinib, Erlotinib) use. The PFS were 8–9 months versus 13–14 months and the OS were 15–26 months versus 28–35 months in patients with and without miliary pulmonary metastases^4,7–9^. Our study reveals shorter TD-TKI and PFS in miliary lung metastasis under Kaplan–Meier method. Univariate analysis by Cox proportional hazard model also identified miliary lung metastasis as significant variables impacting prognosis in TD-TKI and PFS. However, miliary lung metastasis was not a strong prognostic indicator in multivariate analysis by Cox regression model. Other strong prognostic predictors should be considered.

Poor performance status (in TD-TKI, PFS and OS), liver metastasis (in TD-TKI, PFS and OS), and L858R mutation (in OS) were recognized as independent and strong prognostic indicators for poor survival in our study. In prognosis analysis of distant metastasis, Ren et al. recognized liver metastasis as the worst prognostic factor for adenocarcinoma and small cell lung cancer, and Tamura et al. suggested that liver and adrenal gland metastases adversely affect the outcome of NSCLC^13,14^. In EGFR investigation, Zhang et al. and Li et al. revealed more beneficial for exon 19 deletion mutation than L858R point mutation under description of EGFR-TKI^15,16^. With comparison of different TKIs, our study showed that afatinib had significantly better survival than first-generation TKIs in TD-TKI, PFS and OS. LUX-Lung 7 trial revealed longer PFS and time-to-treatment failure under afatinib prescription than under gefitinib, but no significant difference in OS between them. Kim Y et al. also reported that afatinib showed superior PFS data compared with gefitinib or erlotinib^17–19^.

In our study, miliary lung metastasis had higher proportion of poor PS, which had effect on medical decision after PD, such as availability of re-biopsy for T790M mutation evaluation, subsequent chemotherapy, or early intervention of palliative care. Patients with poor PS might have intolerance of second-generation TKI or adequate dosage of medicine due to side effect. Miliary lung metastasis harboring EGFR mutation also implied hematogenous dissemination and strong association with multiple-organ metastasis. Uribe et al. described that EGFR drove metastasis in many ways. Paracrine loops comprising tumor and stromal cells enabled EGFR to fuel invasion across tissue barriers, survival of clusters of circulating tumor cells, as well as colonization of distant organs^20^. Liver metastasis was considered a strong indicator of poor survival with some hypotheses, including hepatic metabolism of TKI and compromise of tumor immunity^21^. Compared with miliary lung metastasis, these prognostic indicators were more dominated in outcome prediction. Besides, further development of third generation of TKI had excellent performance. In FLAURA trial, osimertinib had significantly longer survival compared with first-generation TKI in first-line therapy^22,23^. With the universal availability of osimertinib in clinical practice, the impact on miliary lung metastasis will be expected in the future.

Our study had several limitations. First, this was a retrospective, single-center study which may infer selection bias and misclassification or information bias. Second, the timing of PD may have some deviation because the follow-up timing of chest CT or other radiologic examinations could not be integrated, with period of 2 months to 1 year. Third, the patients diagnosed in 2018 and 2019 may have insufficient time for assessing OS, which may cause censors. Finally, the dosage and frequency of TKIs were varied due to different clinical condition in our real-world setting.

In conclusion, miliary lung metastasis of NSCLC represented higher rate of extra-pulmonary metastasis and EGFR mutation. Miliary lung metastasis also revealed poorer prognosis with borderline statistical significance. However, performance status, liver metastasis, type of EGFR mutation and generation of TKI were more dominant indicators for outcomes evaluation.

## Data Availability

Raw data were generated at the Chi-Mei Medical Center Cancer Registry. Derived data supporting the findings of this study are available from the corresponding author Kuo-Hwa Chiang upon request.

## Abbreviations

NSCLC: Non-small cell lung cancer
MIPC: Miliary intrapulmonary carcinomatosis
EGFR: Epidermal growth factor receptor
TKI: Tyrosine kinase inhibitor
PS: Performance status
TD-TKI: Time to discontinuation of TKI
PFS: Progression-free survival
OS: Overall survival
CI: Confidence interval
CT: Computed tomography
PR: Partial response
SD: Stable disease
PD: Progression disease
NE: Not evaluable
cHR: Crude hazard ratio
aHR: Adjusted hazard ratio
